# Significance of Furin Expression in Thyroid Neoplastic Transformation

**DOI:** 10.3390/cancers15153909

**Published:** 2023-08-01

**Authors:** Maria Teresa Azevedo, Sofia Macedo, Sule Canberk, Luís Cardoso, Tiago Bordeira Gaspar, Ana Pestana, Rui Batista, Manuel Sobrinho-Simões, Paula Soares

**Affiliations:** 1 i3S-Instituto de Investigação e Inovação em Saúde, University of Porto, 4200-135 Porto, Portugal; tazevedo@i3s.up.pt (M.T.A.); amacedo@ipatimup.pt (S.M.); scanberk@ipatimup.pt (S.C.); lcardoso@i3s.up.pt (L.C.); tgaspar@ipatimup.pt (T.B.G.); rbatista@ipatimup.pt (R.B.); ssimoes@ipatimup.pt (M.S.-S.); 2 Institute of Molecular Pathology and Immunology of the University of Porto (Ipatimup), 4200-135 Porto, Portugal; 3 Department of Pathology and Oncology, Faculty of Medicine, University of Porto (FMUP), 4200-139 Porto, Portugal; 4 Abel Salazar Biomedical Sciences Institute (ICBAS), University of Porto, 4050-313 Porto, Portugal; 5 Faculty of Medicine, University of Coimbra, 3000-370 Coimbra, Portugal; 6 Department of Endocrinology, Diabetes and Metabolism, Coimbra Hospital and University Center, 3004-561 Coimbra, Portugal; 7 Charité Comprehensive Cancer Center, Charité-Universitätsmedizin Berlin, 10117 Berlin, Germany; ana.pestana@charite.de; 8 Department of Pathology, Centro Hospitalar de São João, 4200-139 Porto, Portugal

**Keywords:** ACE2, TMPRSS2, Furin, thyroid, thyroid neoplasms

## Abstract

**Simple Summary:**

Angiotensin-Converting Enzyme 2 (ACE2), Transmembrane Serine Protease 2 (TMPRSS2), and Furin are highly expressed in the normal thyroid gland. The putative role played by these molecules in thyroid tumours has not been thoroughly explored to date. Our study shows that the downregulation of *ACE2* mRNA and overexpression of *Furin* mRNA may play a role in thyroid neoplastic transformation. *ACE2* mRNA expression was lower in thyroid neoplasms, as for Furin an increased expression in neoplastic lesions was observed. Furin revealed a high discriminative power between normal adjacent and neoplastic thyroid tissue. Its high expression was significantly correlated with poor prognostic features for thyroid neoplasia.

**Abstract:**

*Angiotensin-Converting Enzyme 2* (ACE2), *Transmembrane Serine Protease 2* (TMPRSS2), and Furin were known to be key players in the SARS-CoV-2 infection, and the thyroid gland was revealed to be one of the relevant targets of the virus. Regardless of the viral infection, the expression of these molecules in the thyroid gland and their putative role in the neoplastic transformation of the thyrocytes has not been thoroughly explored. In this work, we aimed to characterize the mRNA and protein expression pattern of ACE2, TMPRSS2, and Furin in a series of patients with thyroid lesions. Our main results revealed a significantly decreased expression of *ACE2* mRNA in the thyroid neoplasms in comparison to normal adjacent tissue. *Furin* mRNA was significantly increased in thyroid neoplasms when compared to normal adjacent tissue. In addition, a higher *Furin* mRNA level in thyroid carcinomas was associated with the presence of lymph node metastasis. *Furin* mRNA expression revealed a high discriminatory power between adjacent tissue and neoplasms. Protein expression of these molecules did not correlate with mRNA expression. Our study shows the mRNA downregulation of *ACE2* and overexpression of *Furin* in thyroid neoplasms. Further studies are required to clarify if *Furin* expression can be a potential diagnostic indicator in thyroid neoplasia.

## 1. Introduction

Angiotensin-Converting Enzyme 2 (ACE2), Transmembrane Serine Protease 2 (TMPRSS2), and Furin recently gained relevance for being key players in the SARS-CoV-2 infection [1]; the thyroid gland was shown to be one of the targets of the virus [2], and it was advanced that this tropism is related to the mRNA expression levels of these molecules in the gland. 

The ACE2 molecule is found at the surface of cells in many different organs, including the lungs, heart, kidneys, and intestines. It is essential in the regulation of the renin–angiotensin–aldosterone system (RAAS) [3,4,5]. ACE2 converts angiotensin II (Ang II) into angiotensin 1–7, resulting in blood vessel dilation and the lowering of blood pressure, acting as a counterbalance to another enzyme, ACE (Angiotensin-Converting Enzyme). ACE converts angiotensin I (Ang I) into Ang II, whose effect is to constrict blood vessels and raise blood pressure [5,6]. *ACE2* mRNA expression was described to be increased in renal papillary cell carcinoma and colon and pancreatic adenocarcinomas, and decreased in kidney chromophobe, testicular germ cell tumours, and thyroid cancer [7,8,9]. Regarding ACE2 protein expression in cancer, the information is scarce. It is mostly described as expressed in renal and colorectal cancers [8,9,10,11].

The TMPRSS2 protein belongs to the type II transmembrane serine proteases (TTPs) family, which comprise nearly one-third of all proteases [12]. Deregulation of TTP activity is a common characteristic in cancers leading to tumour cell proliferation, invasion, and metastasis [13,14,15,16]. TMPRSS2 plays an important role in the activation of proteins that are involved in blood clotting, and it is implicated in the normal development of the prostate gland [17]. *TMPRSS2* expression was described to be higher in neoplastic and hyperplastic prostate tissue when compared to normal epithelium [18,19]. TMPRSS2 mRNA and protein expression have been described as highly expressed in gastrointestinal tissues (small intestine, stomach, and colon), lung, salivary gland, oesophagus, prostate, liver, and thyroid [10,11,20,21,22,23]. 

Furin is an enzyme that plays an important role in processing several proteins, cleaving proteins at specific sites known as Furin cleavage sites [24,25,26]. These sites are characterized by a specific amino acid sequence, which includes the amino acid sequence Arg-X-(Arg/Lys)-Arg’ [25,27,28]. Furin cleavage is important for the activation of many proteins, including growth factors, receptors, and enzymes [26]. Its overexpression, at the mRNA level, has been associated with several human tumours such as breast [29], head, and neck tumours [30], non-small cell lung carcinoma [31], and ovarian cancer [32], whereas in clear cell renal cell carcinoma and renal papillary carcinoma, the *Furin* mRNA expression was described as relatively low [24].

At the protein level, thyroid cancer has been described as one of the tumours with the highest expression of Furin. Moderate expression was present in lung, liver, prostate, and urothelial cancers [10,11,33]. Some functional studies modulating Furin activity showed the relevance of the protein in the processing of many cancer-related substrates and indicated that high Furin activity promotes the malignant phenotype of cancer cells, namely thyroid cancer cells [34,35].

The expression of these three molecules in abnormal thyroid tissue is not clearly established. Furthermore, the genetic expression of ACE2, TMPRSS2, and Furin has been shown to be altered and implicated in the pathogenesis of several tumours, as previously shown. However, despite a great focus on these molecules in recent years, the data about the roles of ACE2, TMPRSS2, and Furin on the neoplastic transformation of the thyrocytes are still scarce. Therefore, we aim to study the expression of ACE2, TMPRSS2, and Furin in a series of patients with benign and malignant thyroid neoplasms and correlate their expression with clinicopathological features.

## 2. Materials and Methods

### 2.1. Biological Samples

From the Institute of Pathology and Molecular Immunology of the University of Porto (IPATIMUP) medical files, 190 samples of frozen thyroid tissue, corresponding to 165 patients, were collected. The expression patterns of ACE2, TMPRSS2, and Furin in the series of thyroid lesions were characterized by the quantification of the mRNA expression by real-time quantitative PCR (qPCR) that included samples of adjacent thyroid tissue (*n* = 36), goitres (*n*= 2), benign neoplasms, specifically Follicular Thyroid Adenomas (FTAs) (*n* = 76), and malignant neoplasms (*n* = 76) that comprised classical Papillary Thyroid Carcinomas (cPTCs) (*n* = 35), Follicular Variant of Papillary Thyroid Carcinomas (FVPTCs) (*n* = 15), Diffuse Sclerosing Variant of Papillary Thyroid Carcinomas (DSVPTCs) (*n* = 5), Solid Variant of Papillary Thyroid Carcinomas (SVPTCs) (*n* = 1), Oncocytic Variant of Papillary Thyroid Carcinomas (OVPTCs) (*n* = 2), Oncocytic Variant of Papillary Thyroid Carcinomas with other components (*n* = 1), Follicular Thyroid Carcinomas (FTCs) (*n* = 10), Oncocytic Thyroid Carcinomas (OCAs) (*n* = 2), Poorly Differentiated Thyroid Carcinomas (PDTCs) (*n* = 4), and Medullary Thyroid Carcinomas (MTCs) (*n* = 1). 

In addition, the protein expression pattern of these molecules was evaluated in situ by immunohistochemistry (IHC) on a smaller series of 75 formalin-fixed paraffin-embedded (FFPE) thyroid tissues, correspondent to 75 patients. The protein expression was evaluated in the adjacent thyroid tissue, when present (*n* = 60), in goitres (*n* = 5), Grave’s disease (*n* = 1), FTAs (*n* = 15), cPTCs (*n* = 13), FVPTCs (*n* = 19), OVPTCs (*n* = 6), DSVPTCs (*n* = 2), Tall Cell Variant of Papillary Thyroid Carcinomas (TCVPTCs) (*n* = 2), SVPTCs (*n* = 1), Hobnail Variant of Papillary Thyroid Carcinoma (HVPTC) (*n* = 1), FTCs (*n* = 2), OCAs (*n* = 3), and MTCs (*n* = 1). In 4 of the FFPE tissues, only normal adjacent thyroid tissue was available.

### 2.2. Patients’ Characteristics and Clinicopathological Data

The series of patients reported on in this work was partially characterized by Pestana et al. [36]. The clinical and pathological information available for the patients included: age, sex, number of lesions per patient, tumour size, the presence of tumour capsule, capsule invasion/infiltration, lymphovascular invasion, lymph node metastasis, microscopical extrathyroidal extension, lymphocytic infiltrate, and chronic lymphocytic thyroiditis (CLT).

Some of the cases were also previously characterized for TERT mRNA expression and hotspot mutations in BRAF (exon 15), NRAS (exon 2), and TERT (promoter region) [36].

### 2.3. Reverse Transcription of the RNA from Frozen Tissues

For cDNA conversion, 1 µg of total RNA from the thyroid tissue frozen samples was treated with DNase and reverse transcribed using the RevertAid RT Reverse Transcription Kit (REF: K1691, Thermo Fisher Scientific, Waltham, MA, USA) following the manufacturer’s instructions.

### 2.4. Real-Time Quantitative Polymerase Chain Reaction (qPCR)

The mRNA expression of ACE2, TMPRSS2, and Furin was evaluated by qPCR. The PCR reaction was performed with Taqman^®^ Universal PCR Master Mix (REF: 4324018, Applied Biosystems, Foster City, CA, USA). The probes used for the genes of interest were ACE2 qPCR assay (REF: 228806911, IDT, Coralville, IA, USA), TMPRSS2 (REF: 28806907, IDT, Coralville, IA, USA), and Furin (REF: 228806915, IDT, Coralville, IA, USA). The endogenous control Human Tata Box Binding protein (TBP) (REF: 222242490, IDT, Coralville, IA, USA) was used for the normalization of the mRNA expression.

The qPCR method was as follows: an initial step at 95 °C for 10 min, followed by 50 cycles of 15 s at 95 °C and 1 min at 60 °C. Triplicates and non-template controls (NTC) were performed for all the samples. Samples were considered positive when genes were amplified at maximum at cycle threshold (Ct) 37.

### 2.5. Immunohistochemistry

To evaluate the protein expression of ACE2, TMPRSS2, and Furin at the subcellular localization, immunohistochemistry (IHC) was performed in the FFPE samples. First, dewaxing and rehydration of the slides was performed, followed by heat-induced antigen retrieval with EDTA buffer (pH 9.0) using a steamer. The protocol included peroxidase and protein block, antibody amplifier, and polymer incubation with the Ultravision Quanto Detection System HRP (Epredia^®^, Kalamazoo, MI, USA, TL-125-QHL) following the manufacturer’s instructions.

Mouse monoclonal anti-ACE2 (Invitrogen, Waltham, MA, USA, MA5-31395, 1:2500); rabbit monoclonal anti-TMPRSS2 antibody (Abcam, Boston, MA, USA, ab109131, clone EPR3862, 1:1000); and rabbit polyclonal anti-Furin (Invitrogen, PA5-96680; 1:250) were used. The detection was performed with DAB chromogen for all antibodies (Epedria^®^, TA-125-QHDX, Kalamazoo, MI, USA) and slides were counterstained with Gill’s hematoxylin.

### 2.6. Immune Reactive Score (IRS)

Immunohistochemistry evaluation was performed blindly by an endocrine pathologist (S.C.) who evaluated the slides semi-quantitatively using the immunoreactive score (IRS) method [37]. This method consists in the multiplication of the staining intensity score (absent (0), weak (1), moderate (2), and strong (3)) by the percentage of positively stained cells (<10% (0), 10–25% (1), 26–50% (2), 51–75% (3) and>75% (4)). The results obtained ranged from 0 to 12.

Each tissue was analysed entirely. Most of the slides evaluated contained both tumour and adjacent thyroid tissue, and scores were attributed separately.

### 2.7. Statistical Analysis

Statistical analysis and graphical construction were performed using IBM SPSS Statistics 29.0 (IBM, Armonk, NY, USA), GraphPad Prism 8 software version 8.0.2 (GraphPad, San Diego, CA, USA), and Seaborn data visualization library version 0.12.0 [38]. Outlier analysis was performed in both series and only extreme outliers, i.e., values located 3 times the interquartile range value (above the third quartile or below the first quartile) were excluded. The distribution of the data was accessed by Shapiro–Wilk tests. For multiple comparisons, Kruskal–Wallis tests with Bonferroni correction were used, whereas for dichotomic variables, the Mann–Whitney test was applied. Variables such as age and tumour size were categorized according to the cut-off established by the 8th edition of the American Joint Committee on Cancer (AJCC) [39] and median size, respectively (Table 1).

## 3. Results

### 3.1. Clinical Series Characterization

The population of this study was composed of two series; a series of 165 patients bearing thyroid lesions that had RNA extracted from frozen thyroid tissues and a series of 75 patients with FFPE thyroid tissues. The clinicopathological data of the patients is summarized in Table 1.

From the frozen thyroid tissue series, 82.3% of the patients were females (*n* = 130/158), and 17.7% were males (*n* = 28/158). The patients’ median age was 43.0 ± 21 years (median ± IQR), ranging from 11 to 82 years (*n* = 155). Most patients (59.9%) presented a single lesion (*n* = 82/137), and the median tumour size was 3.00 ± 2.50 cm (median ± IQR) (*n* = 130). The majority of the tumours, 66.9% (*n* = 95/142), were encapsulated; 13.8% (*n* = 13/94) had capsule invasion/infiltration, and lymphovascular invasion was present in 20.1% of the cases (*n* = 28/139). Microscopical extra-thyroidal extension (ETE) was observed in 22.5% of the cases (*n* = 23/102), and lymphocytic infiltrate was present in 35.7% of the cases (*n* = 50/140). Information about lymph node metastasis (LNM) was only available for 35 cases, and it was present in almost half of the cases, 48.6% (*n* = 17/35). The mutational status of these tumours was also accessible for *TERTp*, *NRAS,* and *BRAF* genes. Most of the patients were wild-type (WT) for all genes, only 2.6% (*n* = 4/151) presented *TERTp* mutation, 10.5% (*n* = 16/152) presented *NRAS* mutation, and 22.5% (*n* = 20/89) were *BRAF*-mutated; 67.3% (*n* = 107/159) of the cases were positive for *TERT* mRNA expression.

From the FFPE tissue series, 83.1% of the patients were females (*n* = 59/71) and 16.9% males (*n* = 12/71). The patient’s median age was 44 ± 22 years (median ± IQR), ranging from 11 to 76 years (*n* = 71). Most patients presented a single lesion, 68.6% (*n* = 24/35), and the median tumour size was 3.5 ± 2.8 cm (median ± IQR) (*n* = 35). The majority of tumours were encapsulated, 69.7% (*n* = 23/33); capsule invasion occurred in 13.6% (*n* = 3/22), and lymphovascular invasion was present in 26.5% of the cases (*n*= 9/34). Microscopical ETE was observed in 18.2% of the cases (*n* = 4/22), and lymphocytic infiltrate was present in 44.4% of the cases (*n* = 16/36). Half of the cases (*n* = 4/8) had lymph node metastasis. For the hotspot mutations, 2.6% (*n* = 1/38) were mutated for TERTp, 13.5% (*n* = 5/38) were mutated for *NRAS*, and 14.8% (*n* = 4/27) were mutated for *BRAF*. *TERT* mRNA expression was positive in 34.2% of the cases (*n* = 13/38).

### 3.2. mRNA and Protein Analysis in the Thyroid Series

The results obtained from both mRNA and protein expression were stratified and analysed.

1. First, data were analysed by comparing ATs with benign lesions, which included goitres and FTAs, and with carcinomas, which included all malignant thyroid neoplasms;
2. Data were then stratified and separated according to the main histological groups: goitres, FTAs, PTCs, FTCs, OCAs, PDTCs, and MTCs;
3. Finally, all the sub-histological groups were compared: goitres, FTAs, cPTCs, FVPTCs, DSVPTCs, OVPTCs, FTCs, OCAs, and PDTCs.

#### 3.2.1. mRNA Expression in Thyroid Tissues

**Figure 1:**
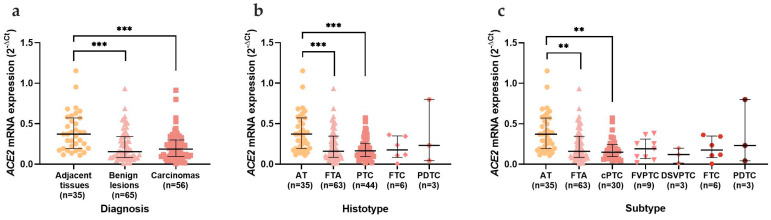
*ACE2* mRNA expression in the thyroid series. Analysis of *ACE2* mRNA expression according to the (**a**) diagnosis; (**b**) histotype; (**c**) subtype. Results are shown as median ± IQR. ** *p*-value ≤ 0.01 and *** *p*-value ≤ 0.001. Abbreviations: AT (Adjacent Thyroid Tissues), FTA (Follicular Thyroid Adenomas), PTC (Papillary Thyroid Carcinomas), FTC (Follicular Thyroid Carcinomas), PDTC (Poorly Differentiated Thyroid Carcinomas), cPTC (classical Papillary Thyroid Carcinomas), FVPTC (Follicular Variant of Papillary Thyroid Carcinomas) and DSVPTC (Diffuse Sclerosing Papillary Thyroid Carcinomas).

**Figure 2:**
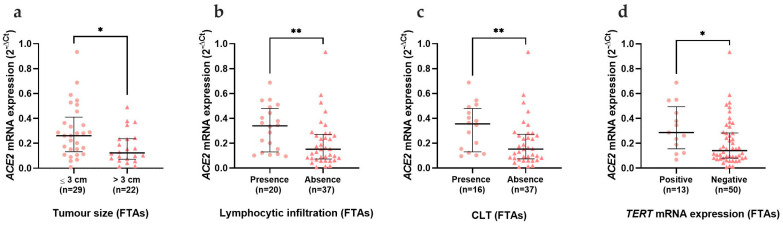
*ACE2* mRNA expression correlation with clinicopathological data. Analysis of *ACE2* mRNA expression in FTAs comparing (**a**) tumour size; (**b**) presence of lymphocytic infiltration; (**c**) presence of CLT and (**d**) *TERT* expression. Results are shown as median ± IQR. * *p*-value ≤ 0.05 and ** *p*-value ≤ 0.01. Abbreviations: CLT (Chronic lymphocytic thyroiditis).

The results concerning *ACE2* mRNA expression revealed a statistically significant decrease (*p* < 0.0001) in benign and malignant thyroid lesions when compared with AT (Figure 1a). When divided according to the histological groups, the decreased expression was still evident, but only significant when comparing ATs to FTAs (*p* = 0.001) and PTCs (*p* < 0.0001) (Figure 1b). The statistical significance of the *ACE2* decrease prevailed for the FTAs (*p* = 0.001) and classical PTCs (*p* = 0.001) when data were divided according to neoplasm subtype (Figure 1c). The clinicopathological data of the series was then compared according to *ACE2* mRNA expression. We observed a significant decrease of *ACE2* in the benign neoplasms that were bigger than 3 cm (*p* = 0.017), with absent lymphocytic infiltration (*p* = 0.005), which corresponded to the absence of CLT (*p* = 0.007) (Figure 2a, 2b and 2c, respectively). *ACE2* was also decreased in follicular adenomas that were negative for *TERT* mRNA expression (*p* = 0.017) (Figure 2d). *ACE2* expression did not reveal any statistically significant differences in the clinicopathological data of the patients with malignant neoplasms.

**Figure 3:**
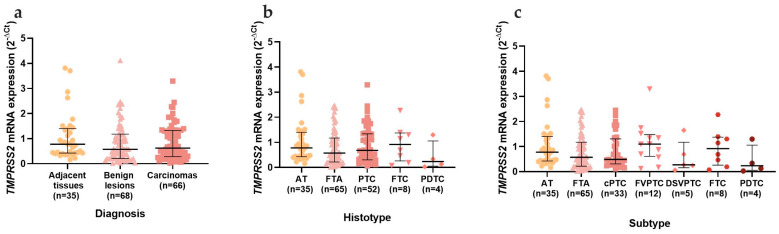
*TMPRSS2* mRNA expression in the thyroid series. Analysis of *TMPRSS2* mRNA expression according to (**a**) diagnosis; (**b**) histotype; (**c**) subtype. Results are shown as median ± IQR. Abbreviations: AT (Adjacent Thyroid Tissues), FTA (Follicular Thyroid Adenomas), PTC (Papillary Thyroid Carcinomas), FTC (Follicular Thyroid Carcinomas), PDTC (Poorly Differentiated Thyroid Carcinomas), cPTC (classical Papillary Thyroid Carcinomas), FVPTC (Follicular Variant of Papillary Thyroid Carcinomas) and DSVPTC (Diffuse Sclerosing Papillary Thyroid Carcinomas).

**Figure 4:**
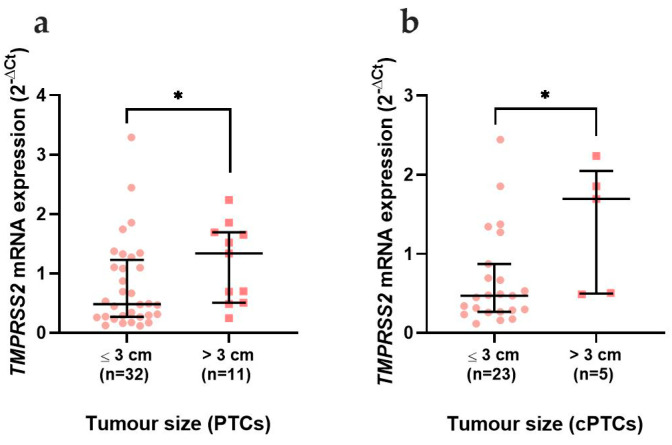
*TMPRSS2* mRNA expression correlation with clinicopathological data. Analysis of *TMPRSS2* mRNA expression in PTCs comparing the: (**a**) tumour size in PTCs and (**b**) tumour size in classical PTCs. Results are shown as median ± IQR. * *p*-value ≤ 0.05. Abbreviations: PTC (Papillary Thyroid Carcinomas) and cPTC (classical Papillary Thyroid Carcinomas).

The mRNA expression of *TMPRSS2,* among the different histotypes and subtypes of thyroid neoplasms, did not show any significant differences (Figure 3). When comparing the patients’ clinicopathological data, *TMPRSS2* was significantly increased (*p* = 0.042) in PTCs that were larger than 3 cm (Figure 4a), a difference that was maintained when only the cPTC (*p* = 0.039) was considered (Figure 4b), despite the low number of cases available.

**Figure 5:**
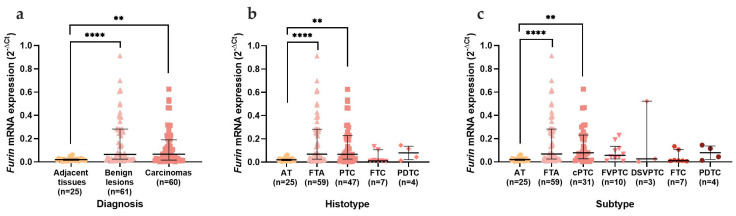
*Furin* mRNA expression in the thyroid series. Analysis of *Furin* mRNA expression according to (**a**) diagnosis; (**b**) histotype; (**c**) subtype. Results are shown as median ± IQR. ** *p*-value ≤ 0.01 and **** *p*-value ≤ 0.0001. Abbreviations: AT (Adjacent Thyroid Tissues), FTA (Follicular Thyroid Adenomas), PTC (Papillary Thyroid Carcinomas), FTC (Follicular Thyroid Carcinomas), PDTC (Poorly Differentiated Thyroid Carcinomas), cPTC (classical Papillary Thyroid Carcinomas), FVPTC (Follicular Variant of Papillary Thyroid Carcinomas) and DSVPTC (Diffuse Sclerosing Papillary Thyroid Carcinomas).

**Figure 6:**
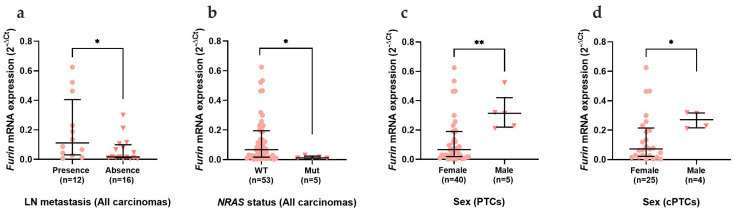
*Furin* mRNA expression and correlation with clinicopathological data. Analysis of *Furin* mRNA expression comparing the (**a**) presence of LNM; (**b**) *NRAS* mutational status in thyroid carcinomas; (**c**) patient sex in PTCs and (**d**) in classical PTCs. Results are shown as median ± IQR. * *p*-value ≤ 0.05 and ** *p*-value ≤ 0.01. Abbreviations: PTCs (Papillary Thyroid Carcinomas) and cPTC (classical Papillary Thyroid Carcinomas).

*Furin* mRNA expression revealed an opposite expression pattern from *ACE2,* since it was significantly increased in benign (*p* < 0.0001) and malignant (*p* = 0.002) thyroid lesions when compared with the adjacent tissue (Figure 5a). In the histotypes category, this increase was significant in adenomas (*p* < 0.0001) and in PTCs (*p* = 0.001) (Figure 5b). The increase in PTCs was mainly due to the classical subtype, which maintained the statistical significance (*p* = 0.002) (Figure 5c). *Furin* revealed statistical differences in the malignant neoplasms of the thyroid. *Furin* was significantly increased in carcinomas with lymph node metastasis (*p* = 0.037) and with wild-type *NRAS* status (*p* = 0.028) (Figure 6a and 6b, respectively). In the PTCs histotype, *Furin* expression was higher in male patients (*p* = 0.002), which was mainly due to the classical PTC subtype (*p* = 0.046) (Figure 6c and 6d, respectively).

#### 3.2.2. ACE2, TMPRSS2, and Furin mRNA Expression Distribution in Adjacent Thyroid Tissue and Thyroid Neoplasms and Furin Discriminative Power

**Figure 7:**
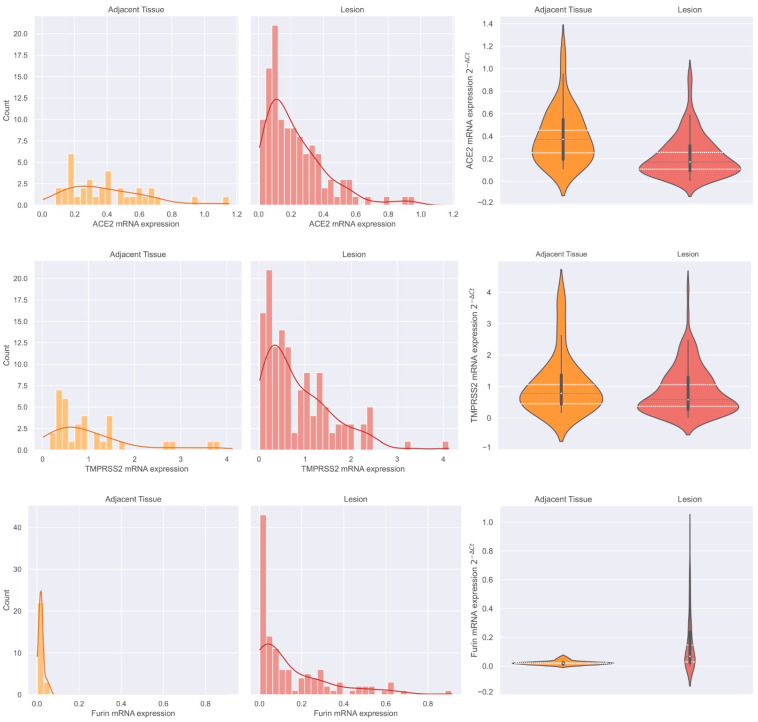
mRNA expression distribution in adjacent thyroid and thyroid neoplasms. Analysis of mRNA expression distribution of *ACE2* (**Top** Panel), *TMPRSS2* (**Middle** Panel), and *Furin* (**Lower** Panel).

To access the distribution of mRNA expression levels of each transcript, the series was divided into two major groups: adjacent thyroid tissues and thyroid neoplasms. The latter includes all lesions present in the series, with the exception of goitres. *ACE2* and *Furin* transcripts showed an unequal distribution of mRNA expression levels. Most thyroid neoplasms had lower *ACE2* expression than adjacent tissue, and the expression levels seemed to be more accumulated in the lower tercile, i.e., the second tercile (2T) of thyroid neoplasms was 0.257 when compared to the 1T of adjacent tissue of 0.253 (Figure 7 Top Panel), whereas *Furin* mRNA expression levels in thyroid lesions seemed to be more accumulated in the third tercile (Figure 7 Lower Panel) compared to the distribution observed in adjacent tissue, i.e., 1T was 0.029 and 2T was 0.023, respectively. Regarding *TMPRSS2* mRNA expression, the distribution was similar between thyroid neoplasms and adjacent normal tissues (Figure 7 Middle Panel).

**Figure 8:**
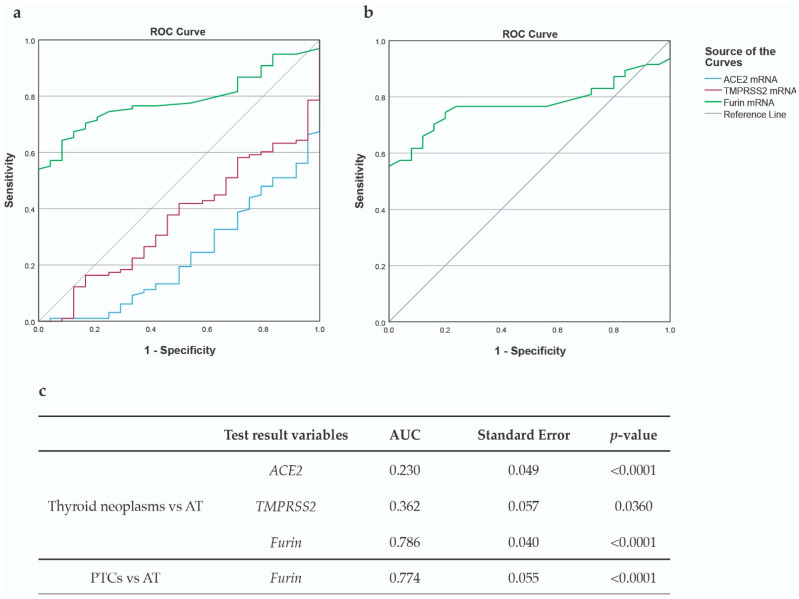
ROC curve analysis. (**a**) AUC representation for *ACE2*, *TMPRSS2*, and *Furin* mRNA expression in adjacent thyroid tissues and thyroid neoplasms; (**b**) *Furin* AUC when data were restricted to PTCs; (**c**) AUC values for *ACE2*, *TMPRSS2,* and *Furin* mRNA expression in both analyses. Abbreviations: AUC (area under the curve).

The ROC curve analysis was performed in order to investigate if any of the transcripts would exhibit any discriminative power in the identification of thyroid neoplasms. In our series, *Furin* stood out as the transcript that presented the highest discriminatory power, displaying an area under the curve (AUC) of 0.786 (Figure 8a,c), thus allowing the identification of thyroid neoplasms with a sensitivity of 70% and a specificity of about 80%. An mRNA expression of *Furin* equal to or greater than 0.025 proved to be the most precise cut-off value for the identification of thyroid neoplasms (Table S1).

A similar relationship was observed when the data were restricted to PTCs, exhibiting an AUC of 0.774 (Figure 8b,c) allowing the identification of this histotype with a sensitivity and a specificity of nearly 80% when the considered cut-off value for *Furin* is equal or greater than 0.0250 (Table S2).

#### 3.2.3. Protein Expression in Thyroid Tissues

**Figure 9:**
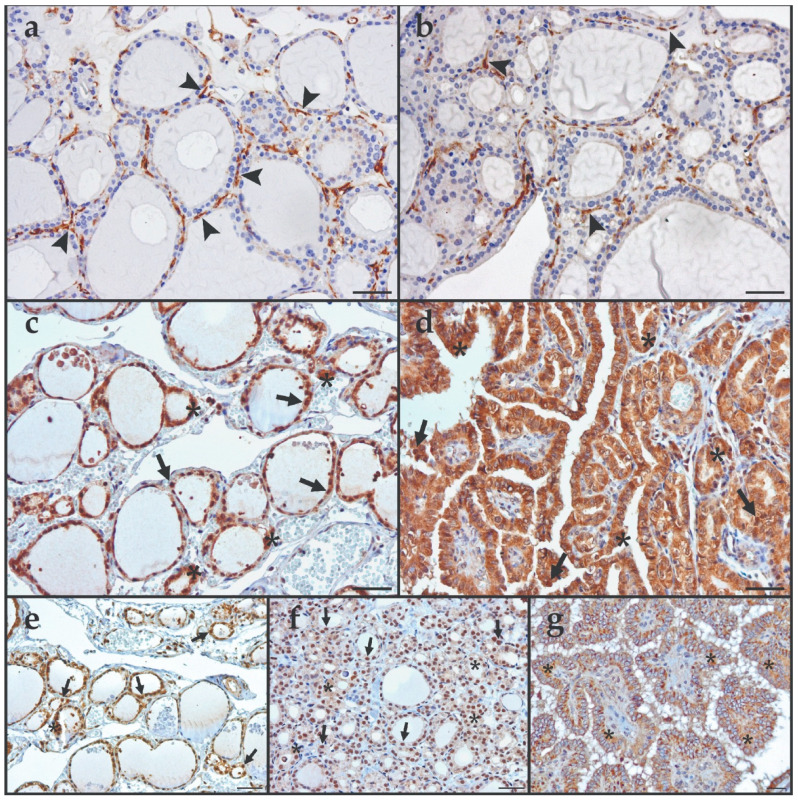
The immunohistochemical staining pattern of ACE2, TMPRSS2, and Furin. Representative images of ACE2 staining in (**a**) adjacent thyroid tissue and (**b**) follicular variant of PTC. The staining is mainly membranous, located in endothelial cells (arrowheads). Representative images of TMPRSS2 staining in (**c**) AT and (**d**) cPTC. The staining is mainly cytoplasmic (asterisks), and occasionally nuclear (arrows), located in follicular cells. Representative images of Furin staining in (**e**) AT, (**f**) FTA, and (**g**) cPTC. The staining is both cytoplasmic (asterisks) and nuclear (arrows), located in follicular cells. Scale bars: 50 μm. Abbreviations: AT (Adjacent Thyroid Tissue), FTA (Follicular Thyroid Adenoma), and cPTC (classical Papillary Thyroid Carcinoma).

The staining pattern observed for ACE2 in thyroid lesions was predominantly membranous and mainly positive in vascular cells surrounding the thyroid follicular cells (Figure 9a,b). No statistically significant differences in protein expression were found among the different groups and categories of the thyroid lesions (*p* > 0.05) (Figure S1). The clinicopathological data did not reveal any statistically significant differences.

In a small number of cases, we were able to detect the ACE2 immunostaining in follicular cells in an apical position, in 12 thyroid neoplasms (2 FTAs and 10 PTCs) and 9 thyroid adjacent tissues. ACE2 apical expression was detected specifically in the papillae of thyroid carcinomas (Figure S2) and was also observed in some tumours with microfollicular histologic patterns.

The staining pattern observed for TMPRSS2 was observed in follicular cells, with a predominantly cytoplasmic and, occasionally, nuclear localization (Figure 9c,d). Cytoplasmic and nuclear staining were analysed separately. No statistical differences were found in TMPRSS2 protein expression among the different histotypes and subtypes of the thyroid lesions, for both cytoplasmic and nuclear expression (Figures S3 and S4). Concerning the clinicopathological data, TMPRSS2 protein expression was higher in males, but only in the adjacent tissue (Figure S5a). In thyroid carcinomas, TMPRSS2 cytoplasmic expression was decreased in the absence of CLT (*p* = 0.031) (Figure S5b), which was also observed, but restricted to the PTC histotype (Figure S5c).

Furin protein expression was predominantly cytoplasmic and occasionally nuclear, localized in follicular cells (Figure 9e–g). No statistically significant differences in the protein expression among the different histotypes and subtypes were detected (*p* > 0.05) (Figure S6). Regarding the clinicopathological data, in thyroid-adjacent tissue, an increase in Furin protein expression was observed in younger patients (<55 years) (Figure S7a) and for the negative *TERTp* mRNA expression (Figure S7b).

## 4. Discussion

The ACE2, TMPRSS2, and Furin molecules became notorious during the SARS-CoV-2 pandemic, since their fundamental role in the mechanism of the virus infection was perceived. This seemed particularly true for the thyroid gland, since this organ is reported to highly express ACE2, *TMPRSS2,* and *Furin* mRNA [24,40,41,42].

Nevertheless, on what concerns ACE2, TMPRSSS2, and Furin expression in thyroid neoplastic lesions, the information available in the literature is scarce. In this work, we aimed to primarily characterize these molecules in a series of thyroid lesions composed of goitres and benign and malignant neoplasms with the intent of understanding their potential role in thyroid carcinogenesis.

The expression of ACE2, TMPRSS2, and Furin was analysed at mRNA and protein levels; the thyroid tissues adjacent to the lesions were also evaluated and used as control.

The first approach was to characterize the mRNA expression of these molecules in the lesions, comparing them with normal adjacent tissues. Regarding the *ACE2* mRNA expression levels, our results showed that benign and malignant thyroid lesions presented significantly decreased expression compared to adjacent thyroid tissue.

Several studies reported high *ACE2* mRNA expression levels in normal thyroid tissues and in thyroid cell lines [43,44,45,46,47]. Our results are consistent with findings in the literature. Bao et al. reported a significant reduction of *ACE2* gene expression in a pooled set of different tumour types, but specifically for thyroid cancer; *ACE2* gene expression was significantly decreased when compared with matched normal tissue [48]. Similarly, Chai et al., using the GEPIA2 tool, reported that thyroid cancer presented decreased *ACE2* expression in comparison to normal tissue [7].

On the other hand, a study encompassing a series of 26 benign and 35 malignant thyroid lesions reported an increase in *ACE2* mRNA expression in malignant lesions such as PTCs and FTCs when compared to FAs and goitres [49]. The authors also observed that undifferentiated thyroid carcinomas presented a decrease in *ACE2* expression when compared to PTCs [49]. In our study, we found lower *ACE2* mRNA in thyroid lesions compared to the normal adjacent tissue, but did not detect significant differences (*p* > 0.05) in *ACE2* mRNA expression between benign and malignant thyroid lesions, since both were diminished in comparison with the normal adjacent tissue.

ACE2’s role in tumorigenesis is still controversial, but several studies claim its association with a more favourable prognosis [7,48,50,51]. A higher expression of *ACE2* predicted better outcomes for disease-free survival and overall survival in tumours such as renal clear cell carcinoma, hepatocellular carcinoma, and ovarian serous cystadenocarcinoma [7]. In breast cancer, patients with higher *ACE2* expression were reported to have longer relapse-free survival [50]. In vitro, ACE2 has also been reported to be involved in tumorigenesis inhibition [50]. In breast cancer cells, *ACE2* expression reduced cell migration and human umbilical vascular endothelial cell proliferation, and downregulated the expression of VEGFa [50]. Cheng et al. reported that *ACE2* overexpression was associated with angiogenesis suppression in non-small cell lung cancer after the development of acquired platinum resistance in human lung cancer xenografts [29].

In our study, we additionally detected, in follicular adenomas, a positive association between *ACE2* expression and smaller tumours (<3 cm) and with the presence of lymphocytic infiltration, which directly correlated with the presence of CLT. Furthermore, *ACE2’s* increased expression was associated with positive *TERT* mRNA expression (*p* < 0.05). These results were concordant with the work by Pestana et al., who also described a significant correlation between *TERT* mRNA expression and lymphocytic presence in smaller tumours [36]. The authors found that most of the FTAs positive for *TERT* mRNA expression presented concomitant lymphocytic thyroiditis, as demonstrated long before [52]. Expression of *ACE2* in lymphocytes has already been described in oral mucosa, the digestive system, and the lungs [53,54,55]; furthermore, *ACE2* has been reported to be positively modulated by cytokines in the thyroid [56]. In the study by Pestana et al., the authors hypothesized that the expression of *TERT* mRNA in FTAs resulted from the presence of lymphocyte infiltration of the tumours [36]. Taking this into account, it is not surprising that there is a concomitant expression of *TERT* and *ACE2* mRNA in tumours with lymphocytic infiltration. No significant differences were observed with *ACE2* expression in malignant neoplasms.

Regarding ACE2 IHC protein expression, the staining was mainly present in small vessels and not specifically in thyroid follicular cells. The fact that ACE2 protein is not expressed by the thyrocytes, but instead by the pericytes of the endothelial cells, has been reported [45]. However, in a few cases, apical expression of the protein was detected in follicular cells of the tumours, specifically in PTC and in tumours with microfollicular patterns. Still, this pattern of expression was a rare event in our series, and to our knowledge, apical expression of ACE2 in the thyroid cells was not described before. We did not find any common feature in these tumours associated with this pattern of expression. 

*TMPRSS2* showed elevated mRNA levels of expression; however, we did not observe significant differences between expression in adjacent tissue and in thyroid lesions. The information available in the literature regarding this transcript is also limited, with a few studies reporting relatively high mRNA expression levels of *TMPRSS2* in normal thyroid and in a follicular thyroid cell line [23,45,47,57]. *TMPRSS2* has been reported to be dysregulated in other tumours, such as prostate and lung adenocarcinomas [17,19,58]. A few in silico studies pointed to a downregulated expression of *TMPRSS2* in thyroid carcinoma compared to healthy thyroid tissues [48,58,59,60]; we were not able to observe this, since according to our data, *TMPRSS2* did not seem to play a role in thyroid tumorigenesis.

Concerning *Furin* expression, contrary to what is reported in the GTex database, in our series, *Furin* mRNA in normal thyroid tissue was virtually negative in all samples. We observed a significant increase in *Furin* mRNA expression in benign and malignant lesions compared to adjacent tissue expression (*p* < 0.001). Interestingly, this difference was more pronounced in FTAs (*p* < 0.0001). Different from *ACE2*, *Furin* mRNA revealed significant associations with malignant lesions, in particular the papillary histotype. *Furin* mRNA was significantly higher in tumours from male patients, tumours that were wild -type for the *NRAS* gene, and tumours that presented lymph node metastasis. Interestingly, these parameters seem to be associated with more aggressive behaviour in thyroid tumours. 

Men with thyroid cancer are considered to have a more guarded prognosis. In a series including more than 60,000 patients with thyroid cancer, the male sex presented significantly more aggressive histological subtypes, independently of the age group, and significantly more advanced disease at presentation [61]. Our results revealed *Furin* to be significantly increased in male patients with Papillary Thyroid Carcinomas (*p* = 0.002). 

Regarding *NRAS* mutation, our results revealed a significantly increased expression of *Furin* in thyroid carcinomas wild-type for the *NRAS* mutation that were mostly Papillary Thyroid Carcinomas. *RAS* mutations in thyroid cancer have generally been associated with less aggressive behaviour, specifically when present in the papillary histotype [62,63]. Given our results and the fact that *Furin* seems to be associated with a more aggressive signature in carcinogenesis of the thyroid, it would be reasonable to hypothesize that *Furin* would not be associated with *NRAS* mutation.

Our study revealed *Furin* to be increased in thyroid cancer patients with lymph node metastasis (*p* < 0.05). *Furin* is increased in several types of cancers, and a recent report shows *Furin* to be significantly associated with more aggressive clinicopathological characteristics and poorer patient outcomes in PTC [34]. In a large cohort of PTC patients, *Furin* was found to be highly expressed in almost 45% of the series. In addition, it was significantly associated with clinicopathological characteristics such as advanced stage, tall cell variant, extrathyroidal extension, and high American Thyroid Association risk score [34]. Furthermore, the authors also report a significant association between *BRAF* mutation and *Furin* expression [34]. These authors also described that *Furin* ectopic expression increased the invasive and migratory potential of a PTC cell line [34], supporting the hypothesis that *Furin* is involved in thyroid tumorigenesis and associated with poor prognostic features.

In our series, we could not identify any association with *BRAF* or other genetic alteration, however, *Furin* was revealed to be the only molecule that could discriminate normal thyroid adjacent tissue from the thyroid neoplasms (AUC = 0.786), with a sensitivity of 72% and a specificity of 79%, assuming a cut-off value of 0.025. These values were similar when the series was restricted to PTCs, differentiating the malignant lesions from normal tissue with an AUC = 0.774; 77% sensitivity, and 76% specificity, assuming the same cut-off value. Our results are consistent with the findings of Poyil et al., who observed a higher expression of Furin protein in PTCs when compared to normal thyroid tissue [34]. These results suggest *Furin* as a possible candidate biomarker in thyroid disease.

Furin immunoexpression did not reveal any significant differences concerning the different thyroid lesions. Only a few studies described Furin protein expression in the thyroid [2] and in thyroid carcinomas, more specifically in PTCs [34,64]. In one of these studies, a higher expression of Furin in metastatic tissue was observed when compared to the primary PTC [34]. In our study, we could not detect such an association, although at the mRNA level, tumours with lymph node metastases showed a significantly higher expression of *Furin* mRNA than tumours without lymph node metastases.

A limitation in the interpretation of some of our results is the lack of correlation between Furin mRNA and protein expression. Discrepancies between mRNA and protein levels have been reported in the literature for several molecules, reflecting biologic and/or technical issues [65,66]. The levels of mRNA can be informative, but not predictive of protein expression, which makes it essential to always evaluate the two parameters.

## 5. Conclusions

Our work aimed at the characterization of ACE2, TMPRSS2, and Furin in thyroid lesions, including benign and malignant lesions.

We are able to disclose that *ACE2* mRNA expression was decreased in neoplasms when compared to normal tissue; it was more associated with benign lesions, rather than malignant ones, being only significantly increased in smaller adenomas and in the presence of lymphocytic infiltrate, and associated with CLT. On the other hand, *Furin* is significantly more expressed in thyroid neoplasms when compared to the normal tissue, and its high expression was correlated with the malignant histotype and clinicopathological parameters such as male sex, lymph node metastasis, and *NRAS* wild-type status.

Our work revealed that *ACE2* and *Furin* are modulated in thyroid tumorigenesis, and in an opposite manner. Further studies with larger series are needed to confirm *Furin’s* potential utility as a candidate biomarker in thyroid neoplasia.

## Figures and Tables

**Table 1:** Patients’ characteristics and clinicopathological data for frozen and FFPE tissue series.

Patient Characteristics	Frozen Tissue	FFPE Tissue
	*n* = 165	*n* =75
**Age** Mean ± SD Median ± IQR	***n*** **= 155**	***n*** **= 71**
	43.4 ± 15.4	42.2 ± 15.4
	43.0 ± 21.0	44.0 ± 22.0
Min–Max	11–82	11–76
**Age** <55 years	***n*** **= 155**	***n*** **= 71**
	121 (78.1%)	58 (81.7%)
≥55 years	34 (21.9%)	13 (18.3%)
**Sex** Female	***n*** **= 158**	***n*** **= 71**
	130 (82.3%)	59 (83.1%)
Male	28 (17.7%)	12 (16.9%)
**Clinicopathological features**	**Frozen tissue**	**FFPE tissue**
	***n*** **= 168**	***n*** **= 75**
**Number of** **lesions**	***n*** **= 137**	***n*** **= 35**
1	82 (59.9%)	24 (68.6%)
>1	55 (40.1%)	11 (31.4%)
**Tumour size (cm)**	***n*** **= 130**	***n*** **= 35**
Mean ± SD	3.3 ± 1.8	3.6 ± 1.7
Median ± IQR	3.0 ± 2.5	3.5 ± 2.8
Min–Max (cm)	0.5–10.0	0.5–7.0
**Tumour size**	***n*** **= 130**	***n*** **= 35**
≤3 cm	77 (59.2%)	16 (45.7%)
>3 cm	53 (40.8%)	19 (54.3%)
**Tumour capsule**	***n*** **= 142**	***n*** **= 33**
Presence	95 (66.9%)	23 (69.7%)
Absence	47 (33.1%)	10 (30.3%)
**Capsule invasion/infiltration**	***n*** **= 94**	***n*** **= 22**
Presence	13 (13.8%)	3 (13.6%)
Absence	81 (86.2%)	19 (86.4%)
**Lymphovascular invasion**	***n*** **= 139**	***n*** **= 34**
Presence	28 (20.1%)	9 (26.5%)
Absence	111 (79.9%)	25 (73.5%)
**Lymph node metastases**	***n*** **= 35**	***n*** **= 8**
Presence	17 (48.6%)	4 (50.0%)
Absence	18 (51.4%)	4 (50.0%)
**Microscopical extrathyroidal extension**	***n*** **= 102**	***n*** **= 22**
Presence	23 (22.5%)	4 (18.2%)
Absence	79 (77.5%)	18 (81.8%)
**Lymphocytic infiltration**	***n*** **= 140**	***n*** **= 36**
Presence	50 (35.7%)	16 (44.4%)
Absence	90 (64.3%)	20 (55.6%)
**Chronic lymphocytic thyroiditis**	***n*** **= 132**	***n*** **= 72**
Presence	43 (32.6%)	13 (18.1%)
Absence	88 (66.7%)	59 (81.9%)
***TERT*** **p**	***n*** **= 151**	***n*** **= 38**
Wild-Type	147 (97.4%)	37 (97.4%)
Mutated	4 (2.6%)	1 (2.6%)
***TERT*** **expression**	***n*** **= 159**	***n*** **= 38**
Positive	107(67.3%)	25 (65.8%)
Negative	52 (32.7%)	13 (34.2%)
***NRAS*** **mutation**	***n*** **= 152**	***n*** **= 38**
Wild-Type	136(89.5%)	33 (86.8%)
Mutated	16 (10.5%)	5 (13.2%)
***BRAF*** **mutation**	***n*** **= 89**	***n*** **= 27**
Wild-Type	69 (77.5%)	23 (85.2%)
Mutated	20 (22.5%)	4 (14.8%)

Note: For some of the characteristics the information was not available in all the cases.

## Data Availability

The data presented in this study are available in this article and supplementary material. Additional information would be available upon reasonable request.

## Supplementary Materials

The following supporting information can be downloaded at: https://www.mdpi.com/article/10.3390/cancers15153909/s1, Table S1. *Furin* mRNA expression cut-off values for all thyroid neoplasms; Table S2. *Furin* mRNA expression cut-off values considering only PTCs; Figure S1. ACE2 immune reactive staining score (IRS) in thyroid series; Figure S2. ACE2 apical staining pattern; Figure S3. TMPRSS2 cytoplasmic IRS score in thyroid series; Figure S4. TMPRSS2 nuclear IRS score in thyroid series; Figure S5. Correlation of TMPRSS2 cytoplasmic IRS with clinicopathological data in adjacent thyroid tissue and carcinomas; Figure S6. Furin cytoplasmic IRS score in thyroid series; Figure S7. Furin IRS score and correlation with clinicopathological data.
