# Supplementary material for: Significance of Furin Expression in Thyroid Neoplastic Transformation
Source: Cancers (Basel). 2023 Aug 1;15(15):3909. doi: 10.3390/cancers15153909 (PMC10417020; doi:10.3390/cancers15153909)
Supplement: Supplementary file 1 [file cancers-15-03909-s001.zip › cancers-2476684-SI.pdf]

Significance of Furin expression in thyroid neoplastic transformation

Supplementary data

Table S1. *Furin* mRNA expression cut-off values for all thyroid neoplasms.

***Furin* mRNA expression ($2^{-\Delta Ct}$) (Positive if greater than or equal to)**	**Sensitivity**	**Specificity**
0.024	74.5%	75.0%
0.025	72.4%	79.2%
0.026	71.4%	79.2%
0.027	70.4%	83.3%

Table S2. *Furin* mRNA expression cut-off values considering only PTCs.

***Furin* mRNA expression ($2^{-\Delta Ct}$) (Positive if greater than or equal to)**	**Sensitivity**	**Specificity**
0.024	76.6 %	72.0 %
0.025	76.6 %	76.0 %
0.027	74.5 %	80.0 %
0.028	72.3%	80.0 %

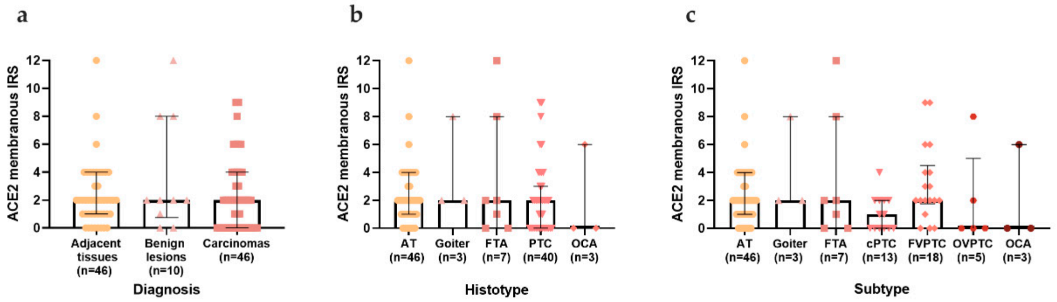

Figure S1. **ACE2 immune reactive staining score (IRS) in thyroid series**. Analysis of ACE2 protein expression score according to the **(a)** Diagnosis; **(b)** Histotype; **(c)** Subtype. Results are shown as median ± IQR. **Abbreviations**: **AT** (Adjacent Thyroid Tissues), **FTA** (Follicular Thyroid Adenomas), **PTC** (Papillary Thyroid Carcinomas), **OCA** (Oncocytic Thyroid Carcinomas), **cPTC** (classical Papillary Thyroid Carcinomas), **FVPTC** (Follicular Variant of Papillary Thyroid Carcinomas) and **OVPTC** (Oncocytic Variant of Papillary Thyroid Carcinoma).

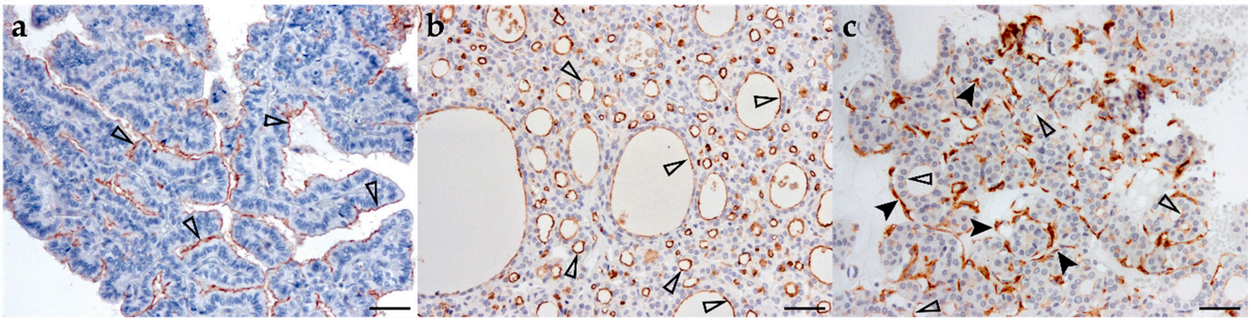

Figure S2. ACE2 apical staining pattern. Representative images of ACE2 apical staining in **(a)** cPTC **(b)** FVPTC and **(c)** a mixed pattern of endothelial (basal) and apical staining in a OVPTC. The apical staining is mainly located in the apical pole of thyrocytes (triangles) surrounded by the basal staining present in endothelial cells (arrowheads). Scale bars: 50 μm. **Abbreviations: FVPTC** (Follicular Variant of Papillary Thyroid Carcinomas) and **OVPTC** (Oncocytic Variant of Papillary Thyroid Carcinoma).

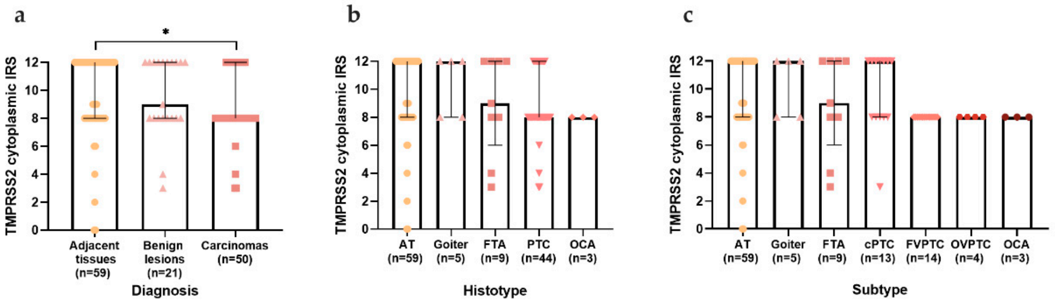

Figure S3. **TMPRSS2 cytoplasmic IRS score in thyroid series**. Analysis of TMPRSS2 cytoplasmic protein expression score according to the **(a)** Diagnosis; **(b)** Histotype; **(c)** Subtype. Results are shown as median ± IQR. **Abbreviations**: **AT** (Adjacent Thyroid Tissues), **FTA** (Follicular Thyroid Adenomas), **PTC** (Papillary Thyroid Carcinomas), **OCA** (Oncocytic Thyroid Carcinomas), **cPTC** (classical Papillary Thyroid Carcinomas), **FVPTC** (Follicular Variant of Papillary Thyroid Carcinomas) and **OVPTC** (Oncocytic Variant of Papillary Thyroid Carcinoma).

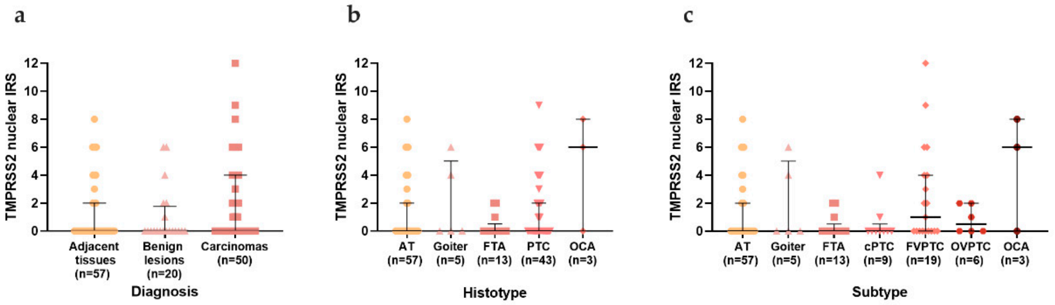

Figure S4. **TMPRSS2 nuclear IRS score in thyroid series**. Analysis of TMPRSS2 nuclear protein expression score according to the **(a)** Diagnosis; **(b)** Histotype; **(c)** Subtype. Results are shown as median ± IQR. **Abbreviations**: **AT** (Adjacent Thyroid Tissues), **FTA** (Follicular Thyroid Adenomas), **PTC** (Papillary Thyroid Carcinomas), **OCA** (Oncocytic Thyroid Carcinomas), **cPTC** (classical Papillary Thyroid Carcinomas), **FVPTC** (Follicular Variant of Papillary Thyroid Carcinomas) and **OVPTC** (Oncocytic Variant of Papillary Thyroid Carcinoma).

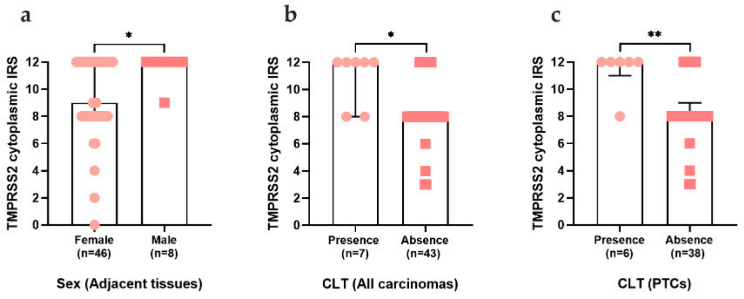

Figure S5. Correlation of TMPRSS2 cytoplasmic IRS with clinicopathological data in adjacent thyroid tissue and carcinomas. Analysis of TMPRSS2 protein expression score comparing the expression on the adjacent tissue according to (a) patient sex; (b) CLT in carcinomas and (c) in PTCs. Results are shown as median ± IQR. **p*-value ≤ 0.05 and ***p*-value ≤ 0.01. **Abbreviations: CLT** (Chronic lymphocytic thyroiditis) and **PTC** (Papillary Thyroid Carcinoma).

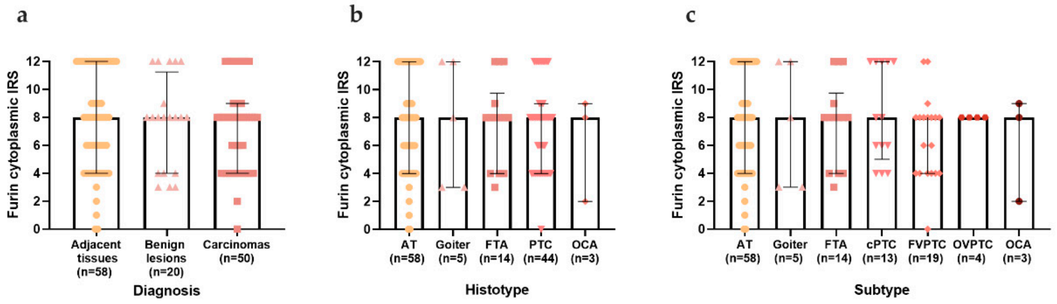

Figure S6. **Furin cytoplasmic IRS score in thyroid series**. Analysis of Furin cytoplasmic protein expression score according to the **(a)** Diagnosis; **(b)** Histotype; **(c)** Subtype. Results are shown as median ± IQR. **Abbreviations**: **AT** (Adjacent Thyroid Tissues), **FTA** (Follicular Thyroid Adenomas), **PTC** (Papillary Thyroid Carcinomas), **OCA** (Oncocytic Thyroid Carcinomas), **cPTC** (classical Papillary Thyroid Carcinomas), **FVPTC** (Follicular Variant of Papillary Thyroid Carcinomas) and **OVPTC** (Oncocytic Variant of Papillary Thyroid Carcinoma).

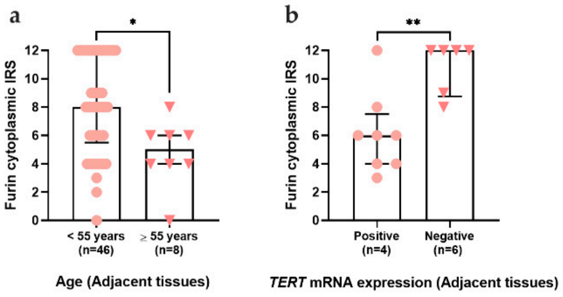

Figure S7. Furin IRS score and correlation with clinicopathological data. Analysis of Furin protein expression score in adjacent tissue comparing the **(a)** Patient age; **(b)** *TERT* mRNA expression. Results are shown as median ± IQR. **p*-value ≤ 0.05 and ***p*-value ≤ 0.01
